# Supplementary material for: The Mental Health and Wellbeing of University Students: Acceptability, Effectiveness, and Mechanisms of a Mindfulness-Based Course
Source: Int J Environ Res Public Health. 2021 Jun 3;18(11):6023. doi: 10.3390/ijerph18116023 (PMC8199969; doi:10.3390/ijerph18116023)
Supplement: Supplementary file 1 [file ijerph-18-06023-s001.zip › ijerph-1249773-supplementary.pdf]

Supplementary Materials

Table S1. Baseline variables predicting completion at post-test and follow-up.

Title	Post-Test		Follow-Up	
	OR	95% CI	OR	95% CI
**Age**	1.11	1.02–1.21	1.06	0.99–1.14
**Gender**				
**Male**	Ref.		Ref.	
**Female**	0.79	0.21–2.97	1.07	0.40–2.89
**Ethnicity**				
**White**	Ref.		Ref.	
**Other**	2.59	0.66–10.18	1.46	0.47–4.52
**Degree level, n (%)**				
**Bachelor's degree**	Ref.		Ref.	
**Master's degree**	2.82	0.47–16.76	0.69	0.21–2.24
**Doctorate**	2.95	0.50–17.70	1.29	0.43–3.90
**Diagnosed Mental Health Problem**				
**no**	Ref.		Ref.	
**yes**	2.22	0.62–7.96	1.67	0.65–4.28
**Currently Mental Health Problem**				
**no**	Ref.		Ref.	
**yes**	3.95	1.07–14.58	3.66	1.33–10.08
**Mental Health Intervention Received**				
**no**	Ref.		Ref.	
**yes**	2.62	0.72–9.51	2.31	0.87–6.12
**Previous experience meditating**				
**no**	Ref.		Ref.	
**yes**	0.21	0.02–2.07	0.71	0.23–2.22
**Expectations for the course**	0.75	0.51–1.08	0.86	0.65–1.14
**WEMWBS**	0.89	0.75–1.07	0.89	0.78–1.03
**CORE-10**	1.12	1.02–1.23	1.07	0.99–1.14
**FFMQ-SF**	0.85	0.76–0.96	0.92	0.86–0.99
**SCS-SF**	0.20	0.06–0.70	0.46	0.22–0.96
**CDRISC**	0.96	0.88–1.05	0.97	0.91–1.04
**Academic goal orientation**				
**Likelihood**	0.97	0.65–1.46	0.92	0.68–1.24
**Skills and resources**	1.10	0.76–1.60	0.88	0.67–1.15
**Commitment**	0.86	0.61–1.21	0.69	0.52–0.92
**Extrinsic motivation**	0.92	0.69–1.23	1.07	0.89–1.29
**Intrinsic motivation**	1.03	0.73–1.44	0.89	0.70–1.14

OR: odds ratio. Ref.: reference category. 95% CI (95% confidence interval). WEMWBS: Warwick Edinburgh Mental Well-being scale; CORE-10: Clinical Outcomes Routine Evaluation-10. FFMQ-SF: Five-Facet Mindfulness Questionnaire-Short Form. SCS-SF: Self-Compassion Scale-Short Form. CDRISC: Connor–Davidson Resilience Scale.

Table S2. Adjusted and imputed analysis of primary outcomes.

Title	Title	Adjusted [a]			Imputed [b]		
**Variable**	**Time**	**d**	**B (95% CI)**	p	d	B (95% CI)	p
WEMWBS	T0						
	T1	0.57	1.98 (1.14 to 2.83)	<0.001	0.61	1.89 (1.65 to 2.12)	<0.001
	T2	0.47	1.97 (1.09 to 2.87)	<0.001	0.53	1.94 (1.44 to 2.44)	<0.001
CORE-10	T0						

	T1	−0.41	−2.29 (−3.60 to −0.99)	<0.001	−0.30	−1.60 (−1.99 to −1.21)	<0.001
	T2	−0.32	−2.03 (−3.40 to −0.65)	0.004	−0.29	−1.76 (−2.60 to −0.92)	<0.001

WEMWBS: Warwick Edinburgh Mental Wellbeing scale. CORE–10: Clinical Outcomes Routine Evaluation–10. [a] Adjusted models include previous experience meditating and treatment expectations as covariates. [b]Imputed models used linear multiple imputations based on chained equations to address missing data at post–test and follow up in the main outcomes, considering: a) those variables included in the primary outcome analysis (e.g. WEMWBS and CORE at baseline); b) variables significantly related or potentially related to non–response (e.g. age, current mental health problem, previous experience meditating, expectations, as well as FFMQ, SCS and commitment at baseline); c) variables that explained a significant amount of variance (r > 0.5) of the main outcomes (e.g. FFMQ, SCS and CDRISC at post–test and at follow–up).

Table S3. Adjusted and imputed analysis of the interaction effect between the time and mental health problems (current or previous) on distress (CORE–10) scores.

Title	Title	**Adjusted [a]**			**Imputed [b]**		
**Mental Health Problems**	**Time**	**d**	**B (95% CI)**	***p***	**d**	**B (95% CI)**	***p***
Current case	T0						
	T1	−0.87	−5.16 (−8.36 to −1.95)	0.002	−0.40	−2.08 (−2.90 to −1.26)	<0.001
	T2	−0.71	−4.23 (−7.64 to −0.83)	0.015	−0.57	−2.95 (−5.07 to −0.83)	0.006
Previous case	T0						
	T1	−0.63	−3.87 (−6.62 to −1.12)	0.006	−0.39	−2.10 (−2.91 to −1.30)	<0.001
	T2	−0.51	−3.14 (−6.02 to −0.27)	0.032	−0.43	−2.32 (−4.08 to −0.56)	0.010

CORE–10: Clinical Outcomes Routine Evaluation–10. [a] Adjusted models include previous experience meditating and treatment expectations as covariates. [b] Imputed models used linear multiple imputations based on chained equations to address missing data at post–test and follow up in the main outcomes, considering: a) those variables included in the primary outcome analysis (e.g. WEMWBS and CORE at baseline); b) variables significantly related or potentially related to non–response (e.g. age, current mental health problem, previous experience meditating, expectations, as well as FFMQ, SCS and commitment at baseline); c) variables that explained a significant amount of variance (r >.5) of the main outcomes (e.g. FFMQ, SCS and CDRISC at post–test and at follow–up).

Table S4. Correlations between the pre–post–treatment changes in the proposed mediators and pre–intervention to follow up changes in the main outcomes (n = 60).

**Process variables / *Outcomes***		**FFMQ–SF**	**SCS–SF**	**CDRISC**	***WEMWBS***	***CORE–10***
**FFMQ–SF**		1				
**SCS–SF**	r (*p*)	0.60 (<0.001)	1			
**CDRISC**	r (*p*)	0.55 (<0.001)	0.44 (<0.001)	1		
**WEMWBS**	r (*p*)	0.51 (<0.001)	0.36 (0.005)	0.32 (0.013)	1	
**CORE–10**	r (*p*)	0.50 (<0.001)	−0.22 (0.096)	−0.30 (0.021)	−0.54 (<0.001)	1

FFMQ–SF: Five–Facet Mindfulness Questionnaire–Short Form. SCS–SF: Self–Compassion Scale–Short Form. CDRISC: Connor–Davidson Resilience Scale. WEMWBS: Warwick Edinburgh Mental Wellbeing scale. CORE–10: Clinical Outcomes Routine Evaluation–10. r: Pearson’s coefficient.
